# The challenges and risk of laboratory handling on a histology specimen during COVID-19 pandemic

**DOI:** 10.1016/j.amsu.2021.102242

**Published:** 2021-03-26

**Authors:** Mohd Fariz Amri, Nornazirah Azizan, Faezahtul Arbaeyah Hussain, Firdaus Hayati, Syed Sharizman Syed Abdul Rahim, Zahir Izuan Azhar

**Affiliations:** a Department of Pathobiology and Medical Diagnostic, Faculty of Medicine and Health Sciences, Universiti Malaysia Sabah, Kota Kinabalu, Sabah, Malaysia; b Department of Pathology, School of Medical Sciences, Health Campus, Universiti Sains Malaysia, Kubang Kerian, Kelantan, Malaysia; c Department of Surgery, Faculty of Medicine and Health Sciences, Universiti Malaysia Sabah, Kota Kinabalu, Sabah, Malaysia; d Department of Community and Family Medicine, Faculty of Medicine and Health Sciences, Universiti Malaysia Sabah, Kota Kinabalu, Sabah, Malaysia; e Department of Public Health Medicine, Faculty of Medicine, Universiti Teknologi MARA (UiTM), Sungai Buloh, Selangor, Malaysia

**Keywords:** Clinical laboratory services, COVID-19, Pathology

## Abstract

The Coronavirus Disease 2019 (COVID-19) pandemic has taken the world into turmoil by surprise. The rapid spreading of this virus has led to an exponential increase in the number of cases. It has created a public health disaster, causing a collapse of the health system in every part of the world. Many sectors in the health area are affected, including histopathology services. The challenges and risk of viral transmission can come from various aspects and levels. For COVID-19 tests, there are even cases of no direct contact with the specimens; the specimens received infection from individuals of unknown status. The fixatives used for histopathology specimens are believed to be inactivated viruses, which can be an inactivate coronavirus. Even so, precautions have to be put in place to prevent the spread of infection to laboratory personnel, especially to those handling underfixed and fresh frozen cytology samples. Precautions must also be taken when dealing with histopathology services, by wearing full personal protective equipment and by executing other standard safety measures. The purpose of this review is to highlight the challenges faced in managing histopathology services in our centre during the COVID-19 pandemic.

## Highlights

• Laboratory personnel are at risk of exposure to the specimens taken from COVID-19 patients.
• The hurdles of receiving fresh frozen section tissue samples due to risk of COVID-19 transmission.
• Sample handling, personal protective equipment, and biosafety level are among the important determinants of safety among laboratory personnel.

## 1 Introduction

In December 2019, an outbreak of pneumonia of uncertain aetiology caught the world by surprise, which originated in Wuhan, Hubei Province, China. The aetiologic agent was initially revealed as Novel Coronavirus 2019, later acknowledged as SARS-CoV-2, and the disease it causes is known as the Coronavirus Disease 2019 (COVID-19) [1]. It has been reported to be linked with a seafood and live-animal market, hypothesising that the infectious agents were initially transmitted from animal-to-human, which subsequently evolved rapidly into a human-to-human transmission. Although still preliminary, current data suggest that bats are the most likely initial source of the SARS-CoV-2 outbreak, which began in Wuhan, China, in December 2019. The outbreak apparently spread from the “wet market” to multiple cities and provinces in China [2]. Since then, it has been rapidly spreading around the world, creating a public health disaster with clinical, psychological, and emotional repercussion, as well as the collapse of the health system and economy that has affected every part of the world. As of 24^th^ August 2020, COVID-19 cases worldwide reached 23 593 443 cases, with 812 626 deaths (5% of the overall cases). Healthcare workers (HCW) are particularly at risk of contracting COVID-19 due to their daily direct or indirect exposure to infected patients, contaminated materials, and surfaces. In the US, 16% of reported COVID-19 cases in April 2020 were among HCW [3]. Infection among HCW in China was higher, up to 29% [4]. As a result of their daily exposure to contaminated samples from positive cases, laboratory personnel are also at risk of contracting COVID-19.

The causative agents, pathogenesis, and clinical features are determinants for the transmission of SARS-CoV-2, a novel beta coronavirus that infects humans and causes an acute respiratory disease [5]. SARS-CoV-2 displays significant similarity (79%) with SARS-CoV based on its nucleotide sequence, its sequence encoding envelope (96%), and its nucleocapsid protein (89.6%) [6]. In contrast, SARS-CoV-2 only shares 50% homology with MERS-CoV.^5^ SARS-CoV and SARS-CoV-2 utilise the angiostensin-converting enzyme-2 receptor for entry into human cells; conversely, MERS-CoV utilises dipeptidyl pepsidase-4 receptor for cellular entry [5]. The clinical features of SARS-CoV-2 infection vary, mostly exhibiting asymptomatic, mild symptoms to acute respiratory distress syndrome and multi-organ failure. Frequently, patients presented fever (not in all), cough, rhinorrhoea, sore throat, headache, and myalgia, whereas a third of patients presented shortness of breath [7]. Conjunctivitis may also occur; thus, it is difficult to distinguish from other more common respiratory infections. In a subset of patients, the disease may progress to pneumonia, respiratory failure, multi-organ dysfunction, and death [7].

## 2 The impact of COVID-19 on anatomic pathology service

First action taken by the nation's health department and the tertiary hospitals involved was by preparing for all possibilities for the COVID-19 to spread out of control; this also included higher education institutions [8]. Such organised and preventive approaches are mandatory to ensure sustainable capability and competency of healthcare personnel, as well as to conserve financial and human resources. Although pale in comparison to the microbiology unit, the anatomic pathology unit is also exposed to SARS-CoV-2, particularly when dealing with fresh tissue and body fluids from patients. Despite the COVID-19 pandemic, laboratory services are essential for patient care, so these services should be maintained as usual. Although the risk of contracting the disease among laboratory personnel is lower than that of other HCW, safety precautions should be put in place to protect them.

Laboratory personnel are among the high-risk workers that have probable exposure to the sample or specimen taken from COVID-19 patients. In fact, this has definitely generated stress, fear, and anxiety among laboratory personnel, medical laboratory technologists, medical officers, and pathologists [9]. In a laboratory environment, sample handling, the use of PPE, and the appropriate biosafety level are among the important determinants that influence the safety of laboratory personnel [10]. This is not only applicable to the molecular diagnostics laboratory that directly deals with COVID-19 specimen, but also applies to other laboratories, including histopathology and cytology.

As the number of elective cases has reduced during this pandemic, most laboratories might experience a decrease in the number of cases. Therefore, this opportunity should be seized to retain only necessary laboratory personnel at any given time and to amend their working shifts. On top of that, the guideline to maintain adequate social distancing among laboratory personnel needs to be established. Should they exhibit any sign of sickness, particularly fever and respiratory symptoms, they are strongly encouraged to seek treatment and must not report for duty. There should be emergency plans in the event that one or more laboratory personnel contract the illness or need to be quarantined, so that an alternate leadership in the laboratory can be appointed.

To control the risk, and as part of the risk assessment, the laboratory staff should be notified by the physician or surgeon each time before submitting a specimen of suspected or confirmed COVID-19 case. This notification can be done through direct communication with the laboratory staff or through proper completion of the request form; it will be best to take both courses of action. Any absence of communication may cause exposure to COVID-19 infection among laboratory workers during the pandemic.

## 3 The impact of COVID-19 on histopathology services

The anatomic pathology unit typically comprises practices of surgical pathology, cytopathology, molecular pathology, and clinical autopsy. Most processes of surgical pathology often involve inactivating many viruses, beginning from putting the tissue specimen into formalin, processing, paraffin embedding, sectioning, and staining [9]. Formalin might have the ability to inactivate coronavirus; however, there is still insufficient evidence to substantiate this. Extra-precaution is still extremely important in handling resected specimens from suspected or positive COVID-19 patients. Additionally, high-risk specimens are recommended to be batched together for handling and processing, and preferably done using separate stations.

When the specimen arrives at the laboratory, only an assigned personnel (wearing full personal protective equipment (PPE)) should attend to the specimen. It is advisable for the specimen to be placed in a 3-layer-seal plastic bag, labelled as biohazard, for the purpose of preventing spillage; the specimen is also to be placed in a carrier box. Precaution should also be taken with regard to the printed that comes along with the specimen. An early study by Hasan et al. showed that there is a potential risk of viral transmission in a paper-based request [10]. Therefore, we have included a new standard of practice for reprinting the request forms of specimens from COVID-19 patients to be done in our laboratory. Formalin has been reported to decrease the infectivity of the virus significantly on day-1, while glutaraldehyde will inactivate COVID-19 after incubation of 1–2 days [11]. Hence, specimens should be handled manually only after formalin has been applied to them for at least 24 h, and this procedure is to be done under the Class II Biological Safety Cabinet. Upon grossing, the medical personnel are advised to change their gloves frequently or disinfect their gloves with 70% ethanol [12].

Several coronaviruses have been found to be non-infectious after exposure to temperatures of 56 °C for 90 min, 67 °C for 60 min, and 75 °C for 30 min [9]. High-temperature paraffin is widely used in the histopathology laboratory. The specimen will be embedded using high-temperature liquid paraffin for cell block preparation. This high-temperature environment during paraffin infiltration inactivates the virus; therefore, formalin-fixed paraffin-embedded (FFPE) tissue blocks are considered to have a low risk of coronavirus infectivity. Despite this, the SARS-CoV-2 RNA extraction can still be detected from the FFPE tissue blocks using RT-PCR technology [13]. Even though histopathology processes might have a low risk of infectivity, it is notable to bear in mind that the frozen section is one of the riskier procedures for laboratory personnel. The frozen section procedure should be avoided to prevent the spread of coronavirus, since it involves aerosol formation; a similar consideration should also be taken in grossing partially fixed specimens [12]. In view of this reason, it is safer to withhold all frozen section activities.

Similar to the frozen section, cytology specimens possess an equal risk. It is better to limit the fine needle aspiration (FNA) procedure during the COVID-19 pandemic since this approach deals with fresh tissue samples. Cytology specimens may contain viable viruses and the processing steps of cytology specimens may lead to the formation of aerosol or droplets [12,14]. However, despite the increased risk of aerosol or droplet exposure, cytopathology services remain essential in the management of patients. FNA for a cytology procedure should only be performed on selected cases by weighing the risks and benefits for the patients. The use of PPE is highly crucial in performing this procedure, and it should be performed in Class II Biosafety Cabinets. The manual handling of cytology samples, such as decapping, sample dilution, vortexing, centrifuging, pipetting, mixing, and preparation for staining on smears, is recommended to be done in a biosafety level-2 laboratory, which should include appropriate physical containment devices, such as a centrifuge with safety buckets or sealed rotors, eye and face protection, double gloves, and a respirator mask [15].

## 4 Conclusion

Laboratory personnel have a risk of exposure to the COVID-19 coronavirus, especially those who are involved either in a patient's care setting, specimen handling, laboratory procedures, FNA procedure, or frozen section evaluation from suspected or infected patients. Thus, using proper PPE, maintaining adequate communication with physicians or surgeons, and proper specimen handling are critically imperative in reducing the risk of infection.

## Source of finance

During this study, no financial or spiritual support was received neither from any pharmaceutical company that has a direct connection with the research subject, nor from a company that provides or provides medical instruments and materials which may negatively affect the evaluation process of this study.

## Authorship contributions

**Idea/Concept**: NA **Design**: MFA; **Control/Supervision**: NA, FH; **Literature Review**: NA, FAH **Writing the Article**: MFA; **Critical Review**: FAH, SSS, AR, ZIA; **References**: FH.

## Ethical approval

Not required.

## Consent

Consents are not required.

## Registration of research studies

Not related.

## Guarantor

Nornazirah Azizan

## Declaration of competing interest

No conflicts of interest between the authors and/or family members of the scientific and medical committee members or members of the potential conflicts of interest, counseling, expertise, working conditions, shareholding and similar situations in any firm.
